# Long-Term Outcomes after Catheter Ablation of Ventricular Tachycardia in Dilated vs. Ischemic Cardiomyopathy

**DOI:** 10.3390/jcm11144000

**Published:** 2022-07-11

**Authors:** Ivaylo Chakarov, Julian Mueller, Elena Ene, Arthur Berkovitz, Kai Sonne, Karin Nentwich, Tobias Schupp, Michael Behnes, Thomas Deneke

**Affiliations:** 1 Clinic for Interventional Electrophysiology, Heart Centre Bad Neustadt, Von-Guttenberg-Straße 11, 97616 Bad Neustadt an der Saale, Germany; ivaylo.chakarov@campus-nes.de (I.C.); elena.ene@campus-nes.de (E.E.); artur.berkovitz@campus-nes.de (A.B.); kai.sonne@campus-nes.de (K.S.); karin.nentwich@campus-nes.de (K.N.); thomas.deneke@campus-nes.de (T.D.); 2 Department of Cardiology and Angiology, Philipps-University Marburg, 35037 Marburg, Germany; 3 First Department of Medicine, University Medical Center Mannheim (UMM), 68167 Mannheim, Germany; tobias.schupp@umm.de (T.S.); michael.behnes@umm.de (M.B.)

**Keywords:** electrical storm, VT ablation, acute heart failure, sudden cardiac death, MACE, mortality, hospitalization

## Abstract

Ischemic (ICM) and dilated cardiomyopathy (DCM) represent the two main underlying heart diseases in patients referred for catheter ablation of ventricular tachycardia (VT). While VT ablation in ischemic cardiomyopathy is relatively well-studied, data in patients with DCM are still scarce. The study aimed to compare the acute and long-term outcomes in patients with ICM and DCM who underwent VT ablation at a high-volume center. Consecutive patients who underwent VT ablation from April 2018 to April 2021 were included retrospectively. Patients with ischemic cardiomyopathy were compared to those with dilated cardiomyopathy. The primary endpoint was rate of VT recurrences, the secondary endpoints included overall mortality, rehospitalization because of cardiac condition (VT, acute heart failure, acute myocardial infarction, heart transplantation or implantation of left ventricular assisting device), and major adverse cardiac events (MACE) at long-term follow-up. A total of 225 patients admitted for first VT ablation were included. A total of 156 patients (69%) revealed ICM and 69 (31%) DCM. After a mean follow-up of 22 months, the primary endpoint of VT recurrence occurred significantly more often in the patients with dilated cardiomyopathy (ICM *n* = 47; 37% vs. DCM *n* = 34; 64%; *p* = 0.001). In regard to the secondary endpoint of overall mortality, there was no difference between the two patient cohorts (DCM *n* = 9; 15% vs. ICM *n* = 22; 16%; *p* = 0.677); the patients with DCM showed significantly higher rehospitalization rates due to cardiac conditions (75% vs. 59%; *p* = 0.038) and more frequent MACE (68% vs. 52%; *p*= 0.036). In a Cox regression model, electrical storm at admission was shown to be a predictor for VT recurrence after successful catheter ablation (HR = 1.942: 95% CI 1.237–3.050; *p* = 0.004), while the ablation of every induced VT morphology during the procedure (HR = 0.522; 95% CI = 0.307–0.885; *p* = 0.016) contributed to a positive long-term outcome. DCM is associated with a higher risk of VT recurrence after catheter ablation compared to ICM. Furthermore, patients with DCM are more frequent re-hospitalized in the majority of cases due the VT recurrence. There is no difference in the long-term mortality between the two cohorts.

## 1. Introduction

Patients with structural heart disease are at increased risk of sudden cardiac death (SCD), mainly due to development of malignant ventricular tachyarrhythmias. The use of implantable cardioverter defibrillators (ICD) significantly reduces the mortality in patients both with ischemic (ICM) and nonischemic cardiomyopathy (NICM) [1,2]. However, ICDs alone cannot prevent the onset of ventricular tachycardias, and the delivered therapy is not always successful. Moreover, ICD shocks have been associated with increased mortality and quality-of-life reduction [3]. The two main therapeutic approaches that have been proven to reduce VT recurrences and consequently the number of delivered ICD therapies are antiarrhythmic drugs (AAD), such as amiodarone, and catheter ablation [4]. The use of amiodarone comes at the cost of proarrhythmic effects and extracardiac toxicity, which may lead to discontinuation of the therapy.

Percutaneous catheter ablation has emerged as a promising therapeutic modality for suppressing VT recurrence by targeting and altering the underlying arrhythmogenic substrate. The rapid advances in ablation catheter technologies and three-dimensional electroanatomic mapping systems in combination with promising data about the safety and efficiency of the procedure have led to indication broadening, thus leading to an increase of the total number of procedures carried out.

A reduction of VT recurrence has been already described after successful catheter ablation for VT, but the outcomes of patients with different underlying cardiac diseases need further investigation [5,6]. In the majority of published reports regarding long-term outcomes after VT ablation, patients were divided based on the presence or absence of coronary artery disease, respectively, into those with ischemic or non-ischemic cardiomyopathy. As NICM represents a group of heterogenous etiologies, each of which is considerably different in their pathophysiological and pathoanatomical phenotypes, we decided to focus on the two clinically most relevant subgroups, namely ICM and dilated cardiomyopathy (DCM). Therefore, we investigated retrospectively the acute and long-term outcomes in patients with ICM and DCM who underwent catheter ablation for VT in our centre.

## 2. Methods

### 2.1. Study Population

We examined retrospectively all consecutive patients who underwent a first catheter ablation for ventricular tachycardia from April 2018 until April 2021 at a tertiary electrophysiological center with established interinstitutional network for patients presenting with VT or ICD shocks. In patients, where more than one ablation procedure was needed during the hospital stay, the first one was considered as index ablation. Patient with ICM based on coronary findings and those with DCM were included and further analyzed in the present study. We define DCM in alignment with the current definition proposed by the European society of cardiology working group on myocardial and epicardial disease [7], which postulates DCM as being marked by left ventricular or biventricular systolic dysfunction and dilatation that are not explained by abnormal loading conditions or coronary artery disease. Patents with other causes of non-ischemic cardiomyopathy, such as congenital heart disease, hypertrophic cardiomyopathy, (sub)acute myocarditis, non-compaction cardiomyopathy, arrhythmogenic right ventricular dysplasia, valvular cardiomyopathy, sarcoidosis, toxic cardiomyopathy, or tachycardia-induced cardiomyopathy, were excluded from the study. Furthermore, all patients with reversible causes for VT (for example, infection, electrolyte disturbances, toxic) were also excluded. All relevant data were extracted from the electronic hospital information system, including discharge letters, ICD interrogation reports, patient files, reports from imaging studies (echocardiography, MRI, CT-scan, coronarography), and reports from the conducted electrophysiological study. The gathered data comprised patient baseline characteristics, prior medical history and medical therapy, detailed laboratory findings, findings from the imaging modalities, as well as data regarding the electrophysiological study.

### 2.2. Electrophysiological Study

Our approach to endocardial and epicardial VT ablation has been described previously [8]. Briefly, written informed consent to the ablation procedure was obtained from all patients prior to the intervention. The majority of the patients underwent conscious sedation with continuous intravenous propofol infusion, with further titration to the desired clinical response and in dependence from the EP protocol. For patients with therapy-resistant cardiogenic shock and those with extracorporeal circulatory support, the procedure was performed under general anesthesia with the assistance of the hospital anesthesiologic team. A non-invasive blood pressure and oxygen saturation monitoring was standard with further invasive blood pressure monitoring and blood gas analysis in case of hemodynamic or respiratory deterioration. The left ventricle was accessed either via transseptal puncture or with retrograde aortic approach. In the cases in which an epicardial origin/substrate was presumed, epicardial access was obtained routinely through anterior epicardial puncture using a Tuohy needle [9]. For VT induction, we used a programmed ventricular stimulation from two different RV sites and one additional LV site with at least two trains of 600–333 ms and up to four extra stimuli decremented to local refractories or 200 ms. In the case of non-inducibility, an additional stimulation under isoprenaline infusion was conducted in some patients. The initial protocol was used after the ablation as control of the ablation success. Twelve-lead ECG recordings of the spontaneous VT or, in the cases when ECG was not available, intracardiac recordings (cycle lengths, far field/near field morphology) from the ICD were compared with the induced VTs in order to identify the clinical VT.

Three-dimensional electroanatomical mapping was performed with the help of CARTO 3 (Biosence Webster Inc., Diamond Bar, CA, USA) or RHYTHMIA (Boston Scientific Corp., Natick, MA, USA) or Ensite Precision (Abbott, St. Paul, MN, USA) mapping system using a multielectrode dedicated mapping catheter (Pentaray, Biosence Webster Inc, Diamond Bar, CA, USA; Advisor HDGrid, Abbott, St. Paul, MN, USA; Intellamap Orion, Boston Scientific Corp., Natick, MA, USA). A high-density voltage map was acquired with areas showing bipolar voltage values of <0.5 mV defined as scar and of >0.5 mV but <1.5 mV as low-voltage areas representing diseased myocardial tissue [10]. Additional sites expressing local abnormal ventricular activities (LAVA) were annotated and further used as guidance for the ablation strategy. Radiofrequency was ubiquitously used as energy source for the ablation using an open irrigated ablation catheter with up to 45 Watt and ablation index value of 700–1000. The endpoint of the catheter ablation was elimination of any inducible VT or all LAVAs in cases of non-inducibility, considered as complete short-term procedure success. Further, a partial success was defined as induction of only non-clinical VTs after ablation and procedure failure as induction of the clinical VT. When epicardial access was used, a solution of 1000 mg cortisone was applied locally for anti-inflammatory purposes, and a drainage was placed for at least 12 h.

### 2.3. Complications

Data regarding periprocedural complication and in-hospital mortality were gathered and further evaluated. As major procedure-associated complications, we considered those related to the vascular or epicardial access, development of complete AV block during the ablation, hemodynamic deterioration with cardiogenic shock during the procedure, pneumonia due to aspiration, as well as stroke.

### 2.4. Definition of Endpoints and Follow-Up

The primary endpoint of the study was VT recurrences. Secondary endpoints comprised time to first hospitalization, overall mortality, and major adverse cardiac events (MACE), defined as the composite of myocardial infarction, stroke, and cardiovascular death. Follow-up period lasted until November 2021. Medical data regarding the endpoints of the study were obtained from the information system of our institution or by direct telephone interview with the patients or their relatives.

### 2.5. Statistical Analysis

Continuous variables are reported as mean ± SD if normally distributed or medians (25th–75th percentile) if not normally distributed. Categorical variables were expressed in absolute and relative frequencies. Variables across the patient groups were compared using either Student’s *t*-test or chi-square/Fischer exact test as appropriate. The VT recurrence rate and mortality were estimated by the Kaplan–Meier model, and the results were compared by log-rank test. Predictors for VT recurrence and all-cause mortality that were considered clinically relevant and have shown an association in a univariate model (*p* < 0.1) were included in a Cox proportional hazard model using a stepwise approach. Hazard ratios (HR) and corresponding 95% confidence intervals (CIs) were presented. All statistical tests were two-sided, and a *p*-value < 0.05 was considered significant.

## 3. Results

### 3.1. Patients and Periprocedural Characteristics

Baseline characteristics of the study population are demonstrated on Table 1. Between April 2018 and April 2021, 69 patients with DCM (65 ± 9 years of age, 87% male, LVEF 32 ± 12%) and 156 patients with ICM (68 ± 11 years of age, 92% male, LVEF 33 ± 12%) who underwent catheter ablation of VT were identified in the database. On admission, antiarrhythmic therapy with amiodaron was more frequently administered in the DCM group; furthermore, other AAD such as those of class I or sotalol were seldom used in both groups. In regard to the implanted devices, a CRT-D was more often implanted in patients with DCM (DCM *n* = 27; 43% vs. ICM *n* = 27; 22%; *p* = 0.005). The clinical VTs in both groups showed comparable cycle lengths. More patients in the DCM group presented with electrical storm (ES), defined as ≥3 appropriate ICD intervention or external defibrillations in the last 24 h (DCM *n* = 33, 49% vs. ICM *n* = 49, 31%; *p* = 0.014). Ablation was predominantly conducted under deep sedation and was rarely performed under full anesthesia and intubation (4% in DCM, 3% in ICM; *p* = 0.655). At the beginning of the procedure, a mean of 1.8 ± 1.5 VTs were induced in patients with DCM and, respectively, a mean of 1.7 ± 1.5 in those with ICM. VT non-inducibility (22% in DCM, 21% in ICM; *p* = 0.835) and not hemodynamically tolerated VTs (35% in DCM, 26% in ICM, *p* = 0.172) were comparably distributed. All patients underwent endocardial mapping and ablation. Further epicardial mapping and ablation was performed in 18 (27%) patients with DCM, whereas only 10 (6%) of the patients with ICM had epicardial ablation (*p* = 0.001). Furthermore, the total procedure time and fluoroscopy time was greater in the DCM group. A complete procedure success with non-inducibility of any VTs was achieved in 48 DCM patients (69%) and 123 ICM patients (79%; *p* = 0.580). Intraprocedural partial success, defined as induction of only non-clinical VTs after ablation, was achieved in 13 patients of the DCM group (20%) and in 18 patients of the ICM group (12%; *p* = 0.101). Lastly, a further induction of the clinical VT, considered as procedure failure, was observed in 3 patients with DCM (4%) and 9 patients with ICM (6%; *p* = 0.130). The periprocedural data are summarized on Table 2.

### 3.2. Procedure Associated Complications

In total, 24 major procedure-associated events were recorded in the study population, with these being more frequent in the DCM cohort (*n* = 12; 16%) than in the ICM cohort (*n* = 12; 8%; *p* = 0.030). One patient in the ICM group died during the hospitalization due to cardiogenic shock 4 days after the ablation procedure, and one patient in the DCM group died intraprocedurally due to cardiogenic shock, with no procedure-related complication being identified. All periprocedural complications are summarized on Table 3.

### 3.3. Primary and Secondary Endpoints

After a mean follow-up of 22.3 ± 9.7 months, VT recurrences were observed significantly more frequently in the DCM cohort (*n* = 34; 64%) in comparison to the ICM cohort (*n* = 47; 37%; *p* = 0.001). At 1 year follow-up, 55% of the patients with DCM and 33% of the patients with ICM experienced at least one episode of ventricular tachycardia. At the end of follow-up, a total of 9 patients with DCM (15%) and 22 patients with ICM (16%) died, resulting in no significant difference of overall mortality between the two cohorts (*p* = 0.677) Of note, an implantation of permanent left ventricular assistance device (LVAD) in one patient and heart transplantation in further one patient were reported, both being from the DCM cohort. Regarding first rehospitalization rates, 41 patients in the DCM group (75%) and 76 patients in the ICM group (59%; *p* = 0.038) had readmission, with further analysis revealing VT recurrence as leading cause in both cohorts, albeit occurring more frequent in the DCM group. Of note, first readmission after index ablation was considered the endpoint, and the cases were censored for further readmission during the follow-up; thus, the data did not represent the overall rehospitalization rate. Furthermore, a significant difference in the rates of major adverse cardiovascular events (MACE) was demonstrated, with the latter also occurring more frequently in the DCM cohort (DCM *n* = 40; 68% vs. ICM *n* = 68; 52%; *p* = 0.036).

**Figure 1:**
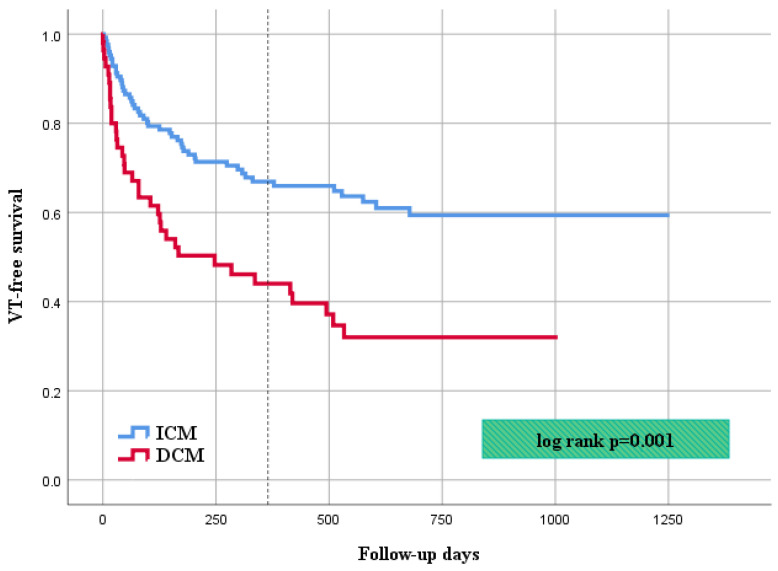
Kaplan–Meier curve showing VT-free survival during follow-up.

**Figure 2:**
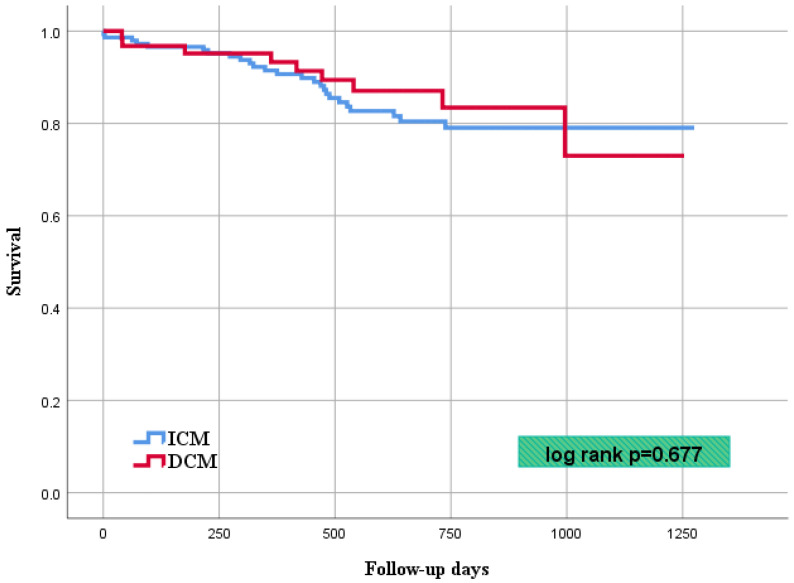
Kaplan–Meier curve showing survival rate after first catheter ablation for VT.

The endpoint results are summarized in Table 4, VT recurrence rate and overall mortality are demonstrated with Kaplan–Meier curves (respectively, Figure 1 and Figure 2).

In Cox regression analysis (Table 5), diabetes mellitus, patients with electrical storm at presentation, amiodaron therapy at discharge, and undergoing epicardial ablation were shown as predictors for VT recurrence during follow-up in the univariate model. On the other hand, complete ablation success (HR = 0.37; 95% CI, 0.24–0.68; *p* = 0.002) was associated with positive outcome at the end of follow-up. In the multivariate model, we managed to show that electrical storm is a strong predictor for VT recurrence such that these patients have two-fold increased risk (HR = 1.94; 95% CI, 1.24–3.05; *p* = 0.004). Protective factors were shown to be the non-inducibility of any VT at procedure end (HR = 0.52; 95% CI, 0.31–0.89; *p* = 0.016). In the separate analysis (Table 6), for each of the cohorts, the complete ablation success emerged again as a strong predictor for VT-free survival in the ICM group, showing a three-fold risk reduction (HR = 0.34; 95% CI, 0.18–0.69, *p* = 0.002). Diabetes mellitus also increased the risk of VT recurrence in the ICM cohort. In the DCM group, none of the included covariates showed statistical significance.

## 4. Discussion

### 4.1. Major Findings

Our study aimed to evaluate and compare the difference in long-term outcomes in patients with ICM and DCM referred for catheter ablation of VT and conveys the following findings:

1. VT recurrence after ablation was more often in patients with DCM such that 64% of the patients experienced at least one sustained VT in the follow-up period;
2. Rehospitalization rate and MACE rate was also higher in patients with DCM, with leading readmissions because of VT recurrence;
3. A significant difference in the overall mortality in the both cohorts during follow-up was not observed;
4. Electrical storm at presentation attributes to higher risk of VT recurrence, while complete ablation success is a predictor for favorable outcome.

The therapy and management of patients with recurrent episodes of ventricular arrythmia is challenging. Catheter ablation has emerged as promising alternative to the use of antiarrhythmic drugs, which has been one the few therapeutic possibilities in the clinical practice. Data on the outcomes of VT ablation in patients with DCM are scarce.

Our study aimed to compare and appreciate the long-term outcomes between patients with dilated and ischemic cardiomyopathy in “real-world” settings. In a population with advanced heart failure and significant left ventricular systolic dysfunction, 36% of the patients with DCM and 63% of the patients with ICM did not experience VT or VF recurrence, which is in line with previously reported data. Achieving non-inducibility of any VT at the procedure end was associated with better long-term results for the whole cohort. Of importance, in the cohort of DCM patients we could not identify any statistically significant predictors for VT recurrence.

To date, four prospective randomized trials have studied the feasibility and safety of catheter ablation in patients with sustained ventricular tachycardia opposed to standard medical therapy [10,11,12,13]. These trials focused explicitly on patients with ischemic cardiomyopathy, with this being a more homogenic population, and managed to prove a prolonged time to first VT recurrence as well as significant reduction in the total numbers of VTs after ablation procedure although without showing any influence of the mortality rate. The VT-free survival after one year of 63% in the ICM cohort is in accordance with data of these studies. A newly published study was the first prospective randomized trial to include patients with non-ischemic cardiomyopathy [14]. The study population consisted of three relatively equally distributed groups with ischemic cardiomyopathy, non-ischemic cardiomyopathy, and arrhythmogenic right ventricular dysplasia and managed to show the feasibility of catheter ablation in reducing the rate of VT recurrence and rehospitalization.

Two large observational studies examined the role of catheter ablation in patients with dilated cardiomyopathy. The observational study of Muser et al. examined 282 patients with non-ischemic dilated cardiomyopathy that underwent endocardial catheter ablation with adjuvant epicardial ablation in 32% of the patients with a median follow-up of 48 months (19–67 months) [15]. The transplant-free survival was 76% and 68% at 60- and 120-month follow-up, respectively, which is in line with our results. The authors showed good long-term outcomes regarding VT-free survival, with 69% of the patients having no VT recurrence after index ablation; moreover, as in our study, the generally younger population with DCM could further profit from discontinuation of the amiodaron therapy.

The HELP-VT study compared retrospectively the short- and long-term outcomes after ablation for ventricular tachycardia comparing patients with ischemic and dilated cardiomyopathy [16]. The authors reported acute complete success of the ablation in 67% of the patients with DCM and 77% in the patients with ICM and achievement of partial success in 22% and 18%, respectively. In our study, we achieved a complete success rate of 76% and 82% in the DCM and ICM cohorts, respectively. Of note, the higher percentage of patients of non-inducible VT in our study should be accounted. In regard to the long-term outcomes at the end of the follow-up period, the cumulative VT-free survival was reported as 43.0% for ICM versus 23.0% for NIDCM with 1-year VT-free survival in ICM was 57% versus 40.5% in NIDCM. In our study population, we have shown a VT-free survival at 1-year follow-up in 45% versus 67% in DCM vs. ICM, respectively. A major difference in our study was the inclusion of patients undergoing first ablation for VT, which may have attributed to the better results. The authors managed to identify both procedure failure and partial success as predictor factors for VT recurrence in the DCM and ICM cohort.

Of importance, DCM is a term describing a phenotypic cardiomyopathy, with different predisposing genetic and acquired etiologies, contributing to the development of the heart disease, so that the patients with DCM are representing a heterogenous population. In a recent study, Ebert et al. examined the incidence and relationship of pathological gene variants in patients with DCM referred for catheter ablation of ventricular tachycardia [17]. The authors reported an 38% incidence of pathogenic carriers associated with development of DCM, mainly LMNA, and showed a two-fold increase of VT recurrence after ablation procedure in those patients compared to patients without genetical predisposition. Furthermore, it is well-recognized that in DCM, the disease progression leads to further myocardial scarring and fibrosis; on the other hand, one can postulate that patients with ICM have a fixed substrate after myocardial infarction. Catheter ablation targets the existing substrate but cannot prevent progression of disease, and thus the unenviable changes occurring in the myocardial substrate may lead to development of new VTs, resulting in higher VT-recurrence rate among DCM patients in our study.

The existing data of the role of non-inducibility after ablation are conflicting. Previous studies have not shown a relationship between VT recurrence and complete ablation success [10,12], while other authors reported that failure to achieve non-inducibility leads to adverse long-term outcome [16,18]. It is reasonable to target all inducible VTs if the clinical context allows it. In our study, achieving complete ablative success in patients with ICM was correlated with positive long-term outcome. In patients with DCM, such correlation was not statistically significant, however there was a trend towards it.

### 4.2. Study Limitations

A mayor limitation is the retrospective character of the study. Furthermore, this is a single-center study reflecting the experience in a large tertiary referral center with operators performing high volume of endo- and epicardial VT catheter ablations and may not be generalized to lower-volume institutions. We decided to include patients undergoing first ablation at our center. Furthermore, after first VT recurrence, the cases were censored, and repeated catheter ablations during follow-up were not further analyzed; thus, the role of a redo procedure cannot be apricated. In some patients, a VT could not be induced at the beginning and at the end of the electrophysiological study, thus making the definition of complete procedure success uncertain.

## 5. Conclusions

Patients with DCM revealed a worse long-term outcome in regard to VT recurrence after catheter ablation compared to patients with ICM. Furthermore, these patients are more frequently re-hospitalized in the majority of cases due the VT recurrence. There is no difference in the long-term mortality between the two cohorts. Elimination of all inducible VTs during the ablation procedure contribute to a long-term VT suppression. Further data are needed to evaluate whether a more aggressive approach may lead to a better control in patients with VT recurrence.

## Figures and Tables

**Table 1:** Baseline characteristics.

Characteristic	DCM (*n* = 69; 31%)		ICM (*n* = 156; 69%)		*p*-Value
Age, median (range)	65 ± 9.2		68 ± 10.8		**0.036**
Males, *n* (%)	60	(87)	143	(92)	0.273
Cardiovascular risk factors, *n* (%)					
Arterial hypertension	55	(80)	146	(94)	**0.001**
Diabetes mellitus	17	(25)	52	(34)	0.182
Hyperlipidemia	41	(59)	129	(83)	**0.001**
Smoking	21	(30)	67	(43)	0.070
Cardiac family history	14	(20)	33	(21)	0.865
Comorbidities, *n* (%)					
Atrial fibrillation	36	(52)	65	(42)	0.144
Stroke	6	(9)	22	(14)	0.251
Chronic kidney disease	35	(51)	95	(61)	0.139
Liver cirrhosis	2	(3)	5	(3)	0.897
COPD	6	(9)	10	(7)	0.556
Asthma	0	(0)	3	(2)	0.245
Medication at admission, *n* (%)					
Beta-blocker	65	(94)	141	(92)	0.585
Amiodarone	34	(49)	44	(29)	**0.003**
Other AAD	2	(3)	1	(1)	0.182
LVEF, %	32 ± 12		33 ± 12		0.375
Type of ICD, *n* (%)					
ICD	36	(57)	95	(74)	**0.005**
CRT-D	27	(43)	27	(22)	
s-ICD	0	(0)	5	(4)	
ICD indication, *n* (%)					
Primary prevention	25	(41)	53	(41)	0.956
Secondary prevention	36	(59)	75	(59)	

AAD, antiarrhythmic drugs; COPD, chronic obstructive pulmonary disease; CRT-D, cardiac resynchronization therapy defibrillator; DCM, dilated cardiomyopathy; ICD, implantable cardioverter defibrillator; ICM, ischemic cardiomyopathy; LVEF, left ventricular ejection fraction; s-ICD, subcutaneous implantable cardioverter defibrillator. Bold values indicate statistical significance.

**Table 2:** Procedural data and intraprocedural success.

Characteristic	DCM (*n* = 69; 31%)		ICM (*n* = 156; 69%)		*p*-Value
Epicardial ablation, *n* (%)	18	(27)	10	(6)	**0.001**
Non-inducible with PES, *n* (%)	15	(22)	32	(21)	0.835
VTs inducible, *n*/patient	1.8 ± 1.5		1.7 ± 1.5		0.618
Clinical VT CL, ms	357 ± 87		361 ± 88		0.788
Procedural duration, min	154 ± 51		134 ± 42		**0.006**
Fluoroscopy duration, min	15.2 ± 11.0		12.3 ± 8.6		**0.050**
Ablation time, min	29.1 ± 19.8		32.4 ± 46.7		0.473
Clinical VT still inducible, *n* (%)	3	(4)	9	(6)	0.130
Any VT inducible, *n* (%)	13	(20)	18	(12)	0.101
Hemodynamic not tolerated VT, *n* (%)	24	(35)	41	(26)	0.172
Catecholamine, *n* (%)	9	(13)	15	(10)	0.408
Intubation, *n* (%)	3	(4)	5	(3)	0.655
Ablation of all VTs, *n* (%)	48	(69)	123	(79)	0.580
Betablocker at discharge, *n* (%)	67	(97)	149	(97)	0.904
Amiodaron at discharge, *n* (%)	26	(38)	37	(24)	**0.039**

CL, cycle length; DCM, dilated cardiomyopathy; ICM, ischemic cardiomyopathy; PES, programmed electrical stimulation; VT, ventricular tachycardia. Bold values indicate statistical significance.

**Table 3:** Complications.

Characteristic	DCM (*n* = 69; 31%)		ICM (*n* = 156; 69%)		*p*-Value
Major complications, *n* (%)	12	(16)	12	(8)	**0.030**
Vascular access related	1	(1)	2	(1)	1.000
Third degree AV block	3	(4)	2	(1)	0.165
Pneumonia	2	(3)	1	(1)	0.223
Cardiogenic shock	1	(1)	3	(2)	1.000
Pneumothorax	1	(1)	2	(1)	1.000
Stroke	0	(0)	1	(1)	1.000
In-hospital mortality, *n* (%)	1	(1)	1	(1)	1.000

AV, atrioventricular; DCM, dilated cardiomyopathy; ICM, ischemic cardiomyopathy. Bold values indicate statistical significance.

**Table 4:** Primary and secondary endpoints.

Characteristic	DCM (*n* = 69; 31%)		ICM (*n* = 156; 69%)		*p*-Value
Primary endpoint, *n* (%)					
VT recurrence	34	(64)	47	(37)	**0.001**
Secondary endpoints, *n* (%)					
First rehospitalization, overall	41	(75)	76	(59)	**0.038**
VT	33	(59)	42	(32)	**0.001**
Acute heart failure	4	(7)	30	(23)	**0.010**
Acute myocardial infarction	2	(4)	0	(0)	0.089
Stroke	1	(2)	2	(1)	1.000
LVAD/HTX	2	(4)	0	(0)	0.089
MACE	40	(68)	68	(52)	**0.036**
Cardiovascular mortality	9	(15)	22	(16)	0.677

DCM, dilated cardiomyopathy; HTX, heart transplantation; ICM, ischemic cardiomyopathy; LVAD, left ventricular assist device; MACE, major adverse cardiac events; VT, ventricular tachycardia. Bold values indicate statistical significance.

**Table 5:** Regression model’s VT recurrence all patients.

	Univariable		Multivariable	
	HR (95% CI)	*p*-Value	HR (95% CI)	*p*-Value
Age	1.003 (0.982–1.025)	0.768	-	-
Diabetes mellitus	1.635 (1.039–2.572)	**0.032**	-	-
Chronic kidney disease	1.235 (0.796–1.917)	0.347	-	-
Electrical storm	2.118 (1.371–3.269)	**0.001**	1.942 (1.237–3.050)	**0.004**
LVEF ≤ 35%	1.224 (0.785–1.909)	0.373	-	-
Partial ablation success	0.741 (0.322–1.705)	0.499	-	-
Complete ablation success	0.374 (0.236–0.667)	**0.002**	0.522 (0.307–0.885)	**0.016**
Epicardial ablation	2.141 (1.222–3.754)	**0.008**	-	-
Amiodaron therapy	1.946 (1.240–3.054)	**0.004**	-	-
Beta blockers therapy	0.433 (0.158–1.183)	0.103	-	-

CI, confidence interval; HR, hazard ratio; LVEF, left ventricular ejection fraction. Bold values indicate statistical significance.

**Table 6:** Regression model VT Recurrence DCM vs. ICM.

	DCM				ICM			
	Univariable		Multivariable		Univariable		Multivariable	
	HR (95% CI)	*p*-Value	HR (95% CI)	*p*-Value	HR (95% CI)	*p*-Value	HR (95% CI)	*p*-Value
Age	0.990(0.955–1.027)	0.603	-	-	1.022 (0.992–1.053)	0.154	-	-
Diabetes mellitus	1.375 (0.644–2.938)	0.410	-	-	2.037 (1.142–3.633)	**0.016**	2.032 (1.134–3.643)	**0.017**
Chronic kidney disease	1.185 (0.610–2.301)	0.617	-	-	1.411 (0.778–2.559)	0.257	-	-
LVEF ≤ 35%	1.625 (0.800–3.302)	0.180	-	-	1.042 (0.584–1.859)	0.889	-	-
Partial ablation success	0.322 (0.095–1.089)	0.068	-	-	1.002 (0.310–3.231)	0.998	-	-
Complete ablation success	0.541 (0.258–1.173)	0.108	-	-	0.342 (0.173–0.677)	**0.002**	0.348 (0.176–0.689)	**0.002**
Epicardial ablation	1.694 (0.829–3.465)	0.148	-	-	1.542 (0.553–4.305)	0.408	-	-
Amiodaron therapy	1.710 (0.873–3.348)	0.118	-	-	1.827 (0.988–3.378)	**0.055**	-	-
Electrical Storm	1.518 (0.779–2.955)	0.220	-	-	2.287 (1.288–4.059)	**0.005**	-	-

CI, confidence interval; DCM, dilated cardiomyopathy; HR, hazard ratio; ICM ischemic cardiomyopathy; LVEF, left ventricular ejection fraction. Bold values indicate statistical significance.

## Data Availability

The data presented in this study are available on request from the corresponding author.
